# Supplementary material for: Social survival: Humpback whales (Megaptera novaeangliae) use social structure to partition ecological niches within proposed critical habitat
Source: PLoS One. 2021 Jun 23;16(6):e0245409. doi: 10.1371/journal.pone.0245409 (PMC8221492; doi:10.1371/journal.pone.0245409)
Supplement: S1 File — (1) Site fidelity, (2) Behavior, (3) Social associations, (4) Social preferences, (5) Network structure & stability. Supplementary tables and figures also included. (PDF) [file pone.0245409.s001.pdf]

S1 File

Social survival: humpback whales (*Megaptera novaeangliae*) use social structure to partition ecological niches within proposed critical habitat

Janie Wray, Eric M. Keen, Éadin N. O'Mahony

Appendix 1. Site fidelity

Appendix 2. Behavior

Appendix 3. Social associations

Appendix 4. Social preferences

Appendix 5. Network structure & stability

Supplementary Tables

Supplementary Figures

Appendix 1. Site fidelity

Detailed account of methods
We characterized population site fidelity based upon the tendency of humpback whales to occupy the study area or to return to it over some period of time [1]. To do so, we used population-level and individual-based metrics of within-season and interannual observations.

Population-level residency patterns were examined using Lagged Identification Rates (LIR; [2,3]). LIR for a particular time lag (τ) is the probability that an individual identified at any time 0 is re-identified τ days hence. Plots of the LIR demonstrate how whales use the area: if the LIR curve falls steeply after time 0, many individuals are leaving the study area after a brief residence period. A LIR falling to zero indicates permanent emigration from the study area in our case (probably not death, as may be the case in shorter-lived species); a LIR that levels off above zero at large τ indicates either permanent residency or re-immigration during the time lags observed [2]. LIR confidence intervals were obtained using 100 bootstrap replicates [3,4]. The maximum τ for this analysis was set to 230 days within the same year as time 0, which was greater than the field season for all years of the study (223 d), and only lags with 10 or more paired identifications were used.

To evaluate the statistical significance of the observed LIR curve, we compared it to a curve generated by a null model in which whales moved in and out of the study area randomly. To generate these null data, we carried out iterative randomizations of the data stream (after Farine [5]), shuffling the identifications collected in each encounter and recalculating the LIR (n=1,000 iterations). We compared the observed LIR curve to the 95% confidence interval of the null model (calculated using the 0.025 and 0.975 quantiles of the null distribution of randomized LIR curves), and interpreted any departure of the observed curve from the confidence interval to represent statistically significant patterns in seasonal residency. In this and all permutation tests below, we confirmed that the sample size of the randomization routine was sufficient to achieve a stable p-value.

To identify the most likely factors affecting LIR dynamics, we used SOCPROG 2.9 [6] to fit exponential decay curves to observed LIRs (see Table S6) using a combination of modeled demographic parameters: population size (N), mean residence time (a), mean time outside the study area (b), emigration (l), immigration (m) and mortality rates (d) (after Whitehead [7] and Perryman *et al.* [3]). Parameters were estimated by maximizing summed log likelihoods (1,000 iterations), and the most parsimonious model was chosen using the quasi-Akaike information criterion (QAIC) instead of AIC due to over-dispersion of the dataset [8,9].

On an individual basis, seasonal occupancy was characterized using standard indicators of site fidelity, including occurrence (*IO*), permanence (*IT*) and periodicity (*It*) [10], as well as the Standardized Site Fidelity Index (SSFI) developed by Tschopp *et al.* [10]. Individual occurrence (*IO*) is the proportion of recaptures, defined as the number of recaptures divided by the number of opportunities for recapture.

$$IO_i = \frac{\sum_{j=1}^{T} c_{ij} - 1}{(T - 1)}$$

Where c_{ij} is a binary value (1=capture, 0=absence or failure to capture) for individual i on sampling occasion j, and T is the number of sampling occasions.

Permanence (*IT*) is the proportion of time spent in the study area, given by the time between the capture and last recapture (F_i) as a proportion of the sampling period (F):

$$IT_i = \frac{F_i}{F}$$

Where F is the time between the first and last sampling occasion. An F_i of 0 indicates that an individual was captured once but never recaptured.

Periodicity (*It*) is the recurrence of an individual, defined as the inverse of the average time between successive recaptures.

$$It_i = \left(\frac{F_i}{\sum_{j=1}^{T} c_{ij} - 1}\right)^{-1}$$

It is set to 0 for an individual who was never recaptured.

Permanence and periodicity were then used to calculate the Standardized Site Fidelity Index (SSFI). The SSFI lends itself to comparison across other feeding and breeding grounds for this species and is robust against irregular survey effort and imperfect detection probability [10]. Both caveats are commonly encountered in long-term marine megafauna monitoring programs, due to the various challenges presented by a marine environment, and must be taken into consideration when choosing fidelity metrics. The SSFI used here is the harmonic mean of Permanence and Periodicity:

$$SSFI = \frac{2}{\frac{1}{IT} + \frac{1}{It}}$$

For each individual, *IO, IT, It*, and *SSFI* were calculated for each year, then the mean and standard deviation were used to summarize the central tendency and consistency of its occupancy patterns.

To characterize interannual site fidelity, we calculated the annual return rate (number of recaptures of whales seen in previous years / total number of captures) for each year of our study (*sensu* Acevedo *et al.* [11]).

We applied a Kruskall-Wallis rank sum test to ask whether site fidelity metrics for known bubble net feeders differed from the remainder of the identified population. We tested whether the LIR of bubble net feeders differed from the remainder of the identified populations using a randomization of the data stream in which the size of the bubble net feeding population was held constant (n=138 whales) but the identities of bubble netters were shuffled. In each of 1,000 iterations, LIRs of bubble net feeders and other whales were recalculated and the latter was subtracted from the former. The distribution of these differences was used as the null model to gauge the significance of the true difference we observed: observations that fell outside of the 95% confidence interval of the null model (based on quantiles) were considered significant.

Additional Results

The earliest arrival we documented was April 2, 2009 (day of year, doy, 92), but the mean date of first observation each year was day 220 (SD=45) (Table S2). In most years, the rate of arrival increased between late June and mid-August (doy 180 – 230). 80% of identified whales tend to be seen by mid-September (doy 260). The final documented arrivals of the season occurred in late October and early November (doy 299 – 315).

On average, the final observation of an identified whale was day 239 (SD=35), and the mean documented occupancy within the fjord system (days between first and last observation) each year was 20 days (SD=25, min=1, max=130) (Table S2). The mean recapture rate within a season was 62% (SD=12%)

(Table 2). Median values of occupancy (IO), permanence (IT), periodicity (It), and the Standardized Site Fidelity Index (SSFI) were, respectively, 0.019, 0.151, 0.145, and 0.158. These distributions were unimodal, and generally higher for whales seen in more than one year (Table S2).

Permutation tests of the Lagged Identification Rate (LIR) curve indicate that humpback whales remain in the study area for 50 days longer than expected if they were conducting random movements in and out of the study area (Fig S6). The most supported movement model that we fit to the LIR curve (Table S6) included parameters for population size, residence time within the study area, residence time outside of the study area, and permanent emigration / mortality (Fig 4). This model was parameterized as follows:

$$\mathrm{R}(\tau) = \left(\frac{e^{-0.0096676\tau}}{26.5852}\right)\left(\frac{1}{5.0846} + \frac{1}{3.117}e^{-\left(\frac{1}{5.0846}+\frac{1}{3.117}\right)\tau}\right)\left(\frac{1}{\frac{1}{5.0846}+\frac{1}{3.117}}\right)$$

The subpopulation of bubble net feeders scored significantly higher ($p < 0.001$) in all site fidelity metrics tested: annual rate of return, seasonal occupancy, permanence, periodicity, Standardized Site Fidelity Index (SSFI), and the mean minimum stay. Bubble netters arrived earlier than other whales ($p < 0.001$), but there was no significant difference in the mean date of final encounter ($p = 0.456$), which is consistent with longer stays in the area. Based on randomization tests, the LIR of bubble net feeders was significantly greater than the remainder of the identified population for time lags of 1 - 70 days (Fig. S3).

Appendix 2. Behavior

Detailed account of methods
The geography of habitat use, group size dynamics, and behavior was explored using randomization tests. Encounters were pooled according to their location, measured as the swimming distance from it to the farthest-inland corner of our study area (Fig. 1), in bins of 10 km, from 0 km to 100 km. For each variable of interest (e.g., bubble-net feeding), the proportion of encounters in which whales exhibited this behavior was noted within each distance bin. To test whether this rate was statistically significant, we used a randomization routine (1,000 iterations) to shuffle the swimming distances of the encounters and recalculate behavior rates. If the observed rate fell outside of the 95% confidence interval of the randomization set (determined using 0.025 and 0.975 quantiles) at some distance into the fjord system, we concluded that the behavior occurred at that distance more frequently than would be expected by random chance. We carried out this test for the following variables: bubble net feeding, other modes of feeding (including those inferred from surface behavior), social activity / posturing, resting / sleeping, the presence of a known mother, the presence of a calf, average group size and calendar day of encounters.

To statistically test the observed differences in (minimum) group size variation between behavior categories (Fig. S5), we built a Poisson generalized linear mixed model (GLMM) with a log link function:

$$y \sim x + (1 \mid id) + (1 \mid year)$$

We chose a GLMM due to the non-normality of our data, an unbalanced study design, as well as the grouping of data by study year [12], and the Poisson distribution was chosen as it is generally used for count data [13]. The model was built to include behavior categories as a fixed effect, whereby this categorical variable contains eight 'treatments' or categories (Fig. S5). The model was built using R package 'lme4' [14] due to the capacity of function glmr() to incorporate crossed random effects. We included individual whale identification, 'id', and study year as crossed random effects because each individual is measured in multiple years and multiple individuals are measured in each year. We define humpback group size as the response variable to behavior type, under the assumption that group size can swell during a bout of cooperative bubble-net feeding.

Additional Results
20% of identified whales were known mothers who brought calves to the area at least once during our study (see further details on calving rates in Wray *et al.* [15]). 40% were observed bubble net feeding at least once during our study. Eight whales (2% of catalog) were observed bubble net feeding alone. All of these whales were seen often (11 – 101 encounters over 5 – 16 years). Individual IDs of these 'solo' bubble netters were: BCY0013, BCZ0136, BCX0375, BCZ0254, BCY0092, CSY0037, CSY0010, CSZ0030.

We took special note of humpback whale interactions with other marine mammals and shoreline vegetation. 26 whales (6% of catalog) were observed associating with sea lions on 33 occasions. Twelve whales were seen with sea lions on more than one occasion, and one whale has been seen with sea lions on three separate occasions (BCY0430; 90 encounters total). Of these 26 sea lion associates, 54% were known mothers (compared to the catalog-wide rate of 20%), and 81% were known bubble net feeders (compare to 40%). Four whales (<1%) have been observed associated with Northern Resident orca. All of these whales (BCY0706, CSY0042, BCY0327, BCY0135) were seen often (encountered 15 – 52 times over 6 – 12 years), and were each seen associated with orca only once. 25 whales (6% of catalog) were observed rolling in shoreline kelp on 25 occasions. No whale was seen kelp-rolling more than once. Of these 25 kelp rollers, 28% were known mothers and 88% were known bubble net feeders (more than twice the population-level bubble net rate of 40%).

Randomization tests indicated that the geography of several aspects of habitat use (Fig. S4) was significantly unlikely under the null hypothesis that whale behaviors were distributed randomly in space and time (Fig. 3). Bubble-net feeding occurred more frequently than expected in the outer channels and less frequently within inner channels; conversely, other feeding modes (including subsurface feeding inferred from surface behaviors) held the opposite pattern. Mean group size was higher than expected in the outer channels and deep within the fjord system. Social activity / posturing occurred at a significantly high rate deep within the fjord system, and less than expected under random chance in other portions of the study area.

Whales were observed resting or sleeping throughout the study area, but this behavior was significantly less common in the outer channels and significantly more common in the center of the fjord system, around Gil Island, but this may be due to extended survey coverage from Whale Point station, where resting/sleeping whales are less likely to go unnoticed. There was no significant pattern to the spatial distribution of known mothers or calves.

Our generalized linear mixed model (GLMM) indicated a significant difference between bubble-net group size and all other behavior categories (Table S1; Fig. S5). Over-dispersion was detected (Pearson squared residuals/residual df=2.948), however for the purposes of this study this model is deemed sufficient. The response variable, minimum group size, has a range of 1 - 17; mean 1.89; sd 1.73; var 2.98. The crossed random effects ('id' and survey year) yielded small standard deviations (0.3659 and 0.000 respectively), as both the number of observations (n=2851) and number of survey years (n=16) were large. Dropping survey year from the model did not alter the magnitude or confidence intervals of fixed effects, and only reduced the AIC from 8394.5 to 8392.5. Diagnostic plots (Fig. S6) demonstrate a good model fit, using a Poisson distribution for count data.

Appendix 3. Stability of associations

Detailed account of methods

We calculated lagged association rates (LARs) using SOCPROG 2.9 and the R package "asnipe" [16] to describe the temporal stability of relationships over time [2]. For these analyses, we subset our catalog to those individuals seen 10 or more times and used the same sampling periods and maximum time lags as in the LIR analysis above. We used LAR rather than standardized LAR because we were confident of identifying most individuals within groups.

We tested whether dyadic stability differs between whales that practice bubble net feeding and those that we have not observed doing so by calculating several LAR curves: one for all groups encountered, a second for groups that contained known bubble net feeders (these groups could also contain whales not known to bubble net), and a third for groups that contained whales not known to bubble net feed.

We evaluated the significance of LAR observations as we did for the LIR analysis described above: by comparing our observations to a null model generated by data stream randomization (n=100 iterations). Also similar to our LIR analysis, we fit conventional decay models to the data in SOCPROG 2.9 in order to identify feasible social processes underlying the temporal stability of social associations (see Table S7).

Additional Results

The mean number of documented associations per individual was 26.2 (Table 3). A total of 4,358 dyadic associations were observed. Six whales were involved in 8 dyadic associations that were observed at least 35 times.

The most supported movement model that we fit to the LAR curve (Table S7) was one with three parameters in which two levels of casual acquaintances, one more stable than the other, occurred within the population (gof = 430.691 (84 d.f.), p<0.0001). This model was parameterized as follows:

$$g(\tau) = 0.73737e^{-2.1826\tau} + (1 - 0.73737)e^{-0.019568\tau}$$

Appendix 4. Social preferences

Detailed account of methods

To disentangle social affinities from associations that may not be driven by social preferences, we used generalized affiliation indices (GAIs) that control for non-social factors (e.g., joint locational preferences, passive overlap in time and/or space, individual gregariousness, etc.) when constructing network weights [17] (Supplementary Appendix 4). GAIs provide an estimate of the strength of social preferences by statistically removing confounding variation due to non-social factors. High positive GAI values indicate affiliation, while negative values indicate avoidance.

Predictor variables used in the calculation of GAIs were the following: joint pairwise gregariousness of dyads (after Godde *et al.* [18], with the correction from Whitehead and James [17] in which the association index of the dyad of interest is removed); geographic overlap, defined as the proportion of years in which both individuals were identified and that they occurred within 15 km of the same swimming distance into the fjord; and temporal overlap, defined as the proportion of years in which both individuals were identified, out of the total number of years in which at least one of the two was identified. The formulation of the latter two variables was adapted from Whitehead and James [17]. We examined the significance of these predictor variables using Multiple Regression Quadratic Assignment Procedure (MRQAP) tests [19] using the functions in the R package 'asnipe' [16] (1,000 iterations). After any non-significant predictors ($p > 0.05$) were removed, Generalized Affiliation Indices were calculated in R by vectorizing the response matrix (association indices) and the predictor matrices and getting the residuals of a linear model with a binomial error structure ("logit" link function; [20]). To acquire GAIs, these residuals were then divided by the denominator of the SRI (after Whitehead and James [17]).

We used data stream randomizations (n=1,000) to examine the significance of observed indices of association (SRI) and affiliation (GAI) for each dyadic association and across the population of whales identified on at least 5 occasions. In each iteration, we shuffled the individuals identified across all encounters and recomputed dyadic co-occurrences first, SRIs second, and joint pairwise gregariousness indices third. We then recalculated the GAI using observed geographic and temporal overlap metrics.

We tested the hypothesis that there were more preferred relationships than expected by determining whether the observed test statistic fell above the 0.95 quantile of the distribution of randomization results. Prevalent social associations and social preferences would be indicated by significantly higher than expected CVs of SRIs and GAIs, respectively. The occurrence of long-term social preferences would be indicated by a significantly higher than expected standard deviation of GAIs [3,17,21]. We conducted these permutation tests for the following subsets of the humpback whale social network: BNF:BNF (ties among known bubble net feeders); Other:Other (ties among other whales); BNF:Other (ties between known bubble net feeders and other whales).

Appendix 5. Network structure & stability

Detailed account of methods

We used significant dyadic associations and affiliations to build social network visualizations in the R package 'igraph' [22]. To determine the structure of the social network, we selected the Louvain clustering algorithm [23] in 'igraph' to allow for multi-level (nested) and/or overlapping communities. To determine whether the observed modularity (Q, i.e., the modularity coefficient of internal clustering of a network as defined in Newman [24]) and number of communities (k) differed from that of a randomly assorted network, we conducted a randomization test in which the observed association indices of dyads were shuffled, the network was reconstructed, and structural metrics were stored (n = 1,000 iterations). The proportion of randomizations whose structure metrics were less than (in the case of Q) or greater than (in the case of k) the realized metrics indicated the statistical significance of the realized network structure.

To examine the inter-annual stability of network structure, we built association networks for a running four-year interval (2004-2007, 2005-2008, …, 2016-2019; n=12) for the population of humpback whales seen on at least five occasions throughout the 15-year study. We then compared these observed networks to null networks based on 1,000 randomizations each of the original sighting records within each four-year interval, to see if the number of community clusters and their modularity changed over time beyond what would be expected from random chance. We also tracked the relative size of communities and the proportion of the population contained within the five largest communities.

Table S1. Generalized linear mixed model fit by maximum likelihood (Laplace Approximation), modelling the minimum group size variance dependent on behavior categories, and accounting for individual identity and sighting year (crossed random effects). Abbreviations: Std. Error – Standard Error; BNF – bubble-net feeding.

Fixed Effects	Estimate	Std. Error	Z value	P-value
BNF (intercept)	1.387	0.032	42.949	< 2e-16 ***
Feeding	-0.896	0.045	-20.046	< 2e-16 ***
Milling	-0.650	0.320	-2.028	0.0426 *
Other	-0.759	0.192	-3.955	7.65e-05 ***
Resting	-0.976	0.123	-7.931	2.17e-15 ***
Robust	-0.950	0.138	-6.899	5.23e-12 ***
Social	-0.487	0.049	-9.929	< 2e-16 ***
Travel	-0.912	0.046	-19.758	< 2e-16 ***

Table S2. Habitat use statistics for the population of humpback whales identified in our study. For each metric, statistics are given for all identified whales (subset > 0), as well as for all whales seen in at more than one year (subset > 1).

Metrics	Subset	Mean	SD	Median	Min.	Max.
Years seen	> 0	3.7	3.8	2	1	16
	> 1	6	3.9	4	2	16
Encounters	> 0	11.4	19.3	3	1	126
	> 1	19	22.7	10	2	126
Earliest first observation (doy)	> 0	200	45	199	92	306
	> 1	183	38	181	110	273
Latest first observation (doy)	> 0	256	42	269	111	315
	> 1	276	26	281	165	315
Average first observation (doy)	> 0	220	37	222	92	306
	> 1	218	29	220	142	281
Average last observation (doy)	> 0	239	35	247	111	306
	> 1	246	24	251	150	290
Average stay (days)	> 0	20	25	7	1	130
	> 1	28	25	24	1	120
Occupancy (IO)	> 0	0.014	0.019	0.007	0.000	0.106
	> 1	0.021	0.020	0.018	0.000	0.106
Permanence (IT)	> 0	0.117	0.151	0.038	0.000	0.753
	> 1	0.171	0.152	0.148	0.000	0.753
Periodicity (It)	> 0	0.069	0.145	0.024	0.000	1.00
	> 1	0.076	0.099	0.046	0.000	0.625
Standardized Site Fidelity Index	> 0	0.095	0.158	0.045	0.000	1.00
	> 1	0.110	0.111	0.084	0.000	0.700
Interannual return rate	> 0	0.231	0.236	0.125	0.062	1.00
(total study)	> 1	0.353	0.246	0.250	0.125	1.00
Interannual return rate	> 0	0.406	0.312	0.286	0.062	1.00
(since year first seen)	> 1	0.564	0.292	0.556	0.125	1.00

Table S3. Summary of indices of association (Simple Ratio Index; SRI) and affiliation (Generalized Affiliation Index; GAI) for humpback whales identified in the Kitimat Fjord System on 5 or more occasions. Summaries are provided for four subsets of dyadic associations within the population: for all dyads (All:All); for dyads of known bubble net feeders (BNF:BNF); for dyads of whales not known to bubble net feed (Other:Other), and for dyadic pairs of known bubble net feeders and other whales (BNF:Other). The sample size of the GAI dataset is lower than that of the SRI analysis due to data gaps in the geographic locations of encounters.

	**All: All**	**BNF: BNF**	**Other: Other**	**BNF: Other**
Dyads used in association analysis (SRI)	19,306	8,128	2,346	8,832
Individuals in each population subset	197	128	69	197
Are strong associations more common than expected? (*test statistic:* CV of SRI)	$p < 0.001$	$p < 0.001$	$p = 0.112$	$p < 0.001$
Percent of non-zero associations that are significant (α=0.05)	11%	8%	35%	17%
Percentage of associations with SRI > 0	14%	23%	5%	7%
Percent of non-zero associations with SRI <= 0.01	13%	16%	1%	9%
Percent of non-zero associations with SRI <= 0.05	90%	90%	72%	91%
Median dyadic SRI	0.000	0.000	0.000	0.000
Median non-zero SRI	0.021	0.019	0.038	0.025
Mean dyadic SRI	0.004	0.006	0.002	0.002
Mean non-zero SRI	0.028	0.026	0.041	0.028
SD dyadic SRI	0.013	0.017	0.010	0.009
SD non-zero SRI	0.024	0.026	0.022	0.018
Maximum dyadic SRI	0.438[1]	0.438[1]	0.111[2]	0.130[3]
Dyads used in affiliation analysis (GAI)	11,339	5,420	1,052	4,867
Individuals in each population subset	158	108	50	158
Are social preferences more common than expected? (*test statistic:* CV of GAI)	p=0.088	p=0.006	p=0.800	p=0.182
Are there more strong preferences than expected? (*test statistic:* SD of GAI)	p=0.088	p=0.057	p=0.924	p=0.202
Proportion of preferences that are negative	87%	80%	94%	92%
Median dyadic GAI	-0.021	-0.015	-0.045	-0.025
Mean dyadic GAI	-0.004	0.002	0.014	0.007
SD dyadic GAI	0.183	0.178	0.254	0.170
Maximum dyadic GAI	9.049[4]	9.049[4]	3.542[5]	4.989[6]

[1] BCX0711-BCX0083 (seen together 71 times)
[2] CSX0037 – CSX0017 (seen together 1 time)
[3] CSZ0017 – CSZ0010 (seen together 3 times)
[4] CSX0061 – CSX0082 (seen together 1 time; SRI=0.111)
[5] CSX0060 – CSY005 (seen together 1 time; SRI=0.071)
[6] BCX0380 – CSY004 (seen together 1 time; SRI=0.0833)

Table S4. MRQAP tests of predictor variables of simple ratio association indices for humpback whales seen 10 or more times (n=137 whales).

Predictor	Partial correlation	MRQAP P-value
Gregariousness	0.130	0.000
Geographic overlap	0.000	0.030
Temporal overlap	0.149	0.000

Table S5. Tests for assortment by tendencies in behavior, habitat use, and site fidelity for social affiliations among the humpback whales of the Kitimat Fjord System. For each trait of interest, Assortativity Coefficients (AC) from the real preference network (positive GAI interpreted as affiliation; negative GAI interpreted as avoidance) are compared to null distributions from random networks (1,000 randomizations) to gauge significance. Bold p-values indicate two-tailed significance at the a=0.05 level; for positive GAIs, p < 0.025 indicates that fewer than 2.5% of randomization ACs were greater than the real AC. For negative GAIs, it indicates that more than 97.5% of randomization ACs were greater than the real AC. For positive GAIs, ACs that are significantly larger than expected indicate assortment by phenotype (i.e., similar whales prefer each other's company). For negative GAIs, ACs that are significantly *larger* than expected indicate that similar individuals avoid each other. ACs that are significantly *smaller* than expected indicate that similar individuals avoid each other less than it would seem from random interactions.

		**Social preferences (GAI)**					
		Positive (affiliation)			Negative (avoidance)		
**Category**	**Trait**	*Real AC (SE)*	*Random mean (SD)*	*P-value*	*Real AC (SE)*	*Random mean (SD)*	*P-value*
Behavior / Biology	Bubble net rate	0.358 (0.107)	-0.134 (0.359)	0.075	-0.045 (0.009)	-0.008 (0.004)	**0.000**
	Known bubble netter	0.236 (0.302)	-0.121 (0.465)	0.236	-0.002 (0.010)	-0.008 (0.004)	0.900
	Feeding rate	0.687 (0.057)	-0.177 (0.370)	**0.007**	-0.026 (0.010)	-0.008 (0.004)	**0.001**
	Social rate	-0.481 (0.309)	-0.154 (0.359)	0.795	-0.024 (0.012)	-0.008 (0.004)	**0.001**
	Resting rate	0.240 (0.345)	-0.128 (0.387)	0.201	-0.018 (0.008)	-0.008 (0.005)	0.028
	Known mother	-0.706 (0.262)	-0.124 (0.475)	0.820	-0.010 (0.008)	-0.008 (0.004)	0.332
Habitat use	Mean fjord position	0.475 (0.117)	-0.175 (0.351)	0.040	-0.031 (0.009)	-0.008 (0.004)	**0.000**
	SD fjord position	-0.581 (0.189)	-0.144 (0.380)	0.865	-0.023 (0.010)	-0.008 (0.004)	**0.004**
Site fidelity	Years seen	-0.051 (0.138)	-0.143 (0.441)	0.414	-0.013 (0.010)	-0.008 (0.005)	**1.00**
	SSFI	0.041 (0.173)	-0.126 (0.350)	0.334	-0.009 (0.009)	-0.008 (0.004)	0.412
	Average arrival date	-0.425 (0.510)	-0.150 (0.403)	0.698	-0.024 (0.012)	-0.008 (0.004)	**0.001**
	Minimum stay	0.286 (0.149)	-0.137 (0.375)	0.147	-0.006 (0.011)	-0.008 (0.004)	**0.994**

Table S6. Fit of candidate exponential decay models of Lagged Identification Rates (LIR) for humpback whales encountered within the Kitimat Fjord System, ranked by lowest quasi-Akaike Information Criterion. Fit metrics were reported within SOCPROG 2.9 (see SOCPROG documentation for the exact model formulae). ΔQAICc reports the difference between the QAICc of each model and that of the best fitting model.

Model parameters included	Parameters	ΔQAICc	Interpretation
Population size<br>Residence time in<br>Residence time out<br>Permanent emigration / mortality	4	59,252	Strong support
Population size<br>Residence time in<br>Residence time out	3	11 (59,263)	No support
Closed emigration<br>Reimmigration	4	11 (59,263)	No support
Population size<br>Mean residence time in	2	103 (59,355)	No support
Permanent emigration/mortality	1	103 (59,355)	No support
Emigration<br>Reimmigration<br>Permanent emigration / mortality	4	107 (59,359)	No support
Population size	1	800<br>(60,052)	No support
Closed	0	800<br>(60,052)	No support

Table S7. Fit of candidate exponential decay models of Lagged Association Rates (LAR) for humpback whales encountered within the Kitimat Fjord System, ranked by lowest quasi-Akaike Information Criterion. Fit metrics were reported within SOCPROG 2.9 (see SOCPROG documentation for the exact model formulae). ΔQAICc reports the difference between the QAICc of each model and that of the best fitting model.

Biological interpretation	Parameters	ΔQAICc	Interpretation
Two levels of casual acquaintances	3	0 (5,222.0)	Strong support
Rapid dissociation + two levels of casual acquaintances	4	1.9 (5,223.9)	Borderline support
Rapid dissociation, preferred companions, casual acquaintances	3	4.7 (5,226.7)	No support
Rapid dissociation + casual acquaintances	2	6.5 (5,228.5)	No support
Preferred companions + casual acquaintances	2	171.1 (5,393.1)	No support
Rapid dissociation + pref. companions	1	244.8 (5,466.8)	No support
Casual acquaintances	1	2,319.5 (7,541.5)	No support
Null	0	181,166.7 (186,388.7)	No support

Table S8. P-values indicating the relationships among association network position metrics (based on SRIs) and strategies of behavior, habitat use, and site fidelity in the population of humpback whales in the Kitimat Fjord System. P-values represent the proportion of randomizations for which the slope coefficient of the regression (Position metric ~ Trait) that is greater than the real observed value. Based on a two-tailed test with α=0.05, P-values below 0.025 (bold) indicate that the relationship between the position metric and the trait is significantly stronger than expected. P-values (also bold) that are greater than 0.975 indicate that the relationship is significantly weaker than expected.

		**Individual network position metric**			
**Category**	**Trait**	Degree centrality	Betweenness centrality	Closeness centrality	Contacts
Behavior / Biology	Known bubble netter	**0.000**	**0.000**	**0.000**	**0.000**
	Bubble net rate	**0.000**	**0.002**	**0.000**	**0.000**
	Feeding rate	**1.00**	0.199	0.578	**1.000**
	Social rate	0.845	**0.002**	**0.025**	0.855
	Resting rate	**0.997**	0.95	0.995	0.934
	Known mother	0.101	0.589	0.682	**0.003**
Habitat use	Mean fjord position	**0.000**	**0.010**	**0.000**	0.030
	SD fjord position	0.078	**0.000**	**0.002**	0.131
Site fidelity	Years seen	**0.000**	**0.000**	**0.000**	**0.000**
	SSFI	0.270	0.691	**0.001**	0.498
	Average arrival date	0.970	0.107	0.347	0.971
	Minimum stay	**0.001**	0.184	0.239	**0.000**

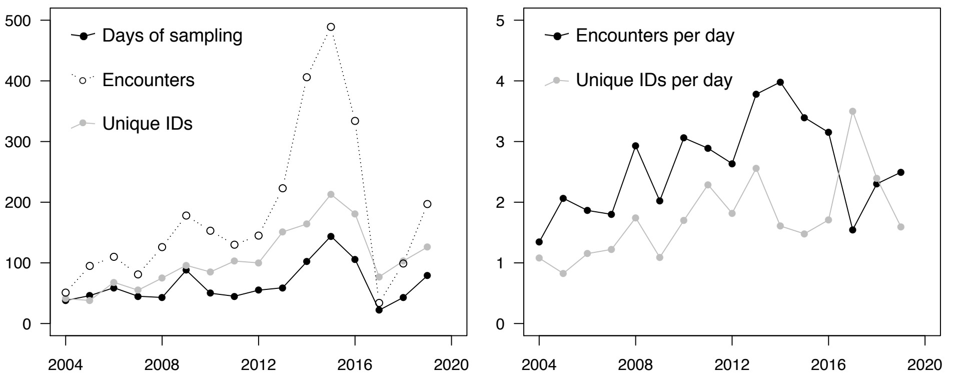

Figure S1. Photo-identification sampling effort and catalog size over the course of this study, 2004-2019. *Left:* Annual totals for the days of sampling (black, solid dots), the number of humpback whale encounters (black, open dots), and the number of unique identifications collected (grey). *Right*: Annual rates for the number of encounters per day of sampling effort (black) and the number of unique identifications collected per day of sampling effort (grey).

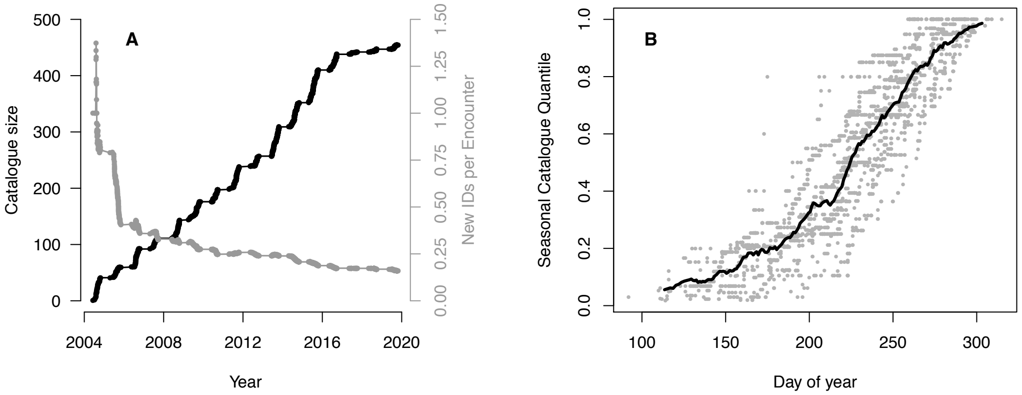

Figure S2. Discovery curves of photo-identified humpback whales in the Kitimat Fjord System, 2004-2019. A: Cumulative size of catalog (black) and catalog growth rate (grey), defined as the number of new identifications obtained per encounter. Right: Annual discovery curve for years 2004-2019, scaled by the size of the annual catalog. Grey dots indicate the proportion of an annual catalog that has been collected as of a given day of year. Black line is a 10-day running average (produced using R package igraph).

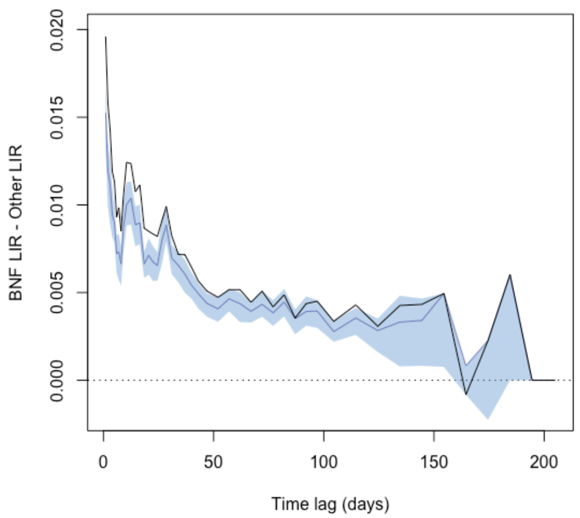

Figure S3. Results of randomization test for the LIR difference between bubble net feeders and the remainder of the population. Black line is the observed LIR difference (BNF – Other). Blue line and shaded area represent the median and 95% confidence interval (2.5% and 97.5% quantiles), respectively, of the permutation tests (n=100). Lags at which the observed LIR rises above the shaded area indicates significant differences in residency behavior between bubble netters and other whales.

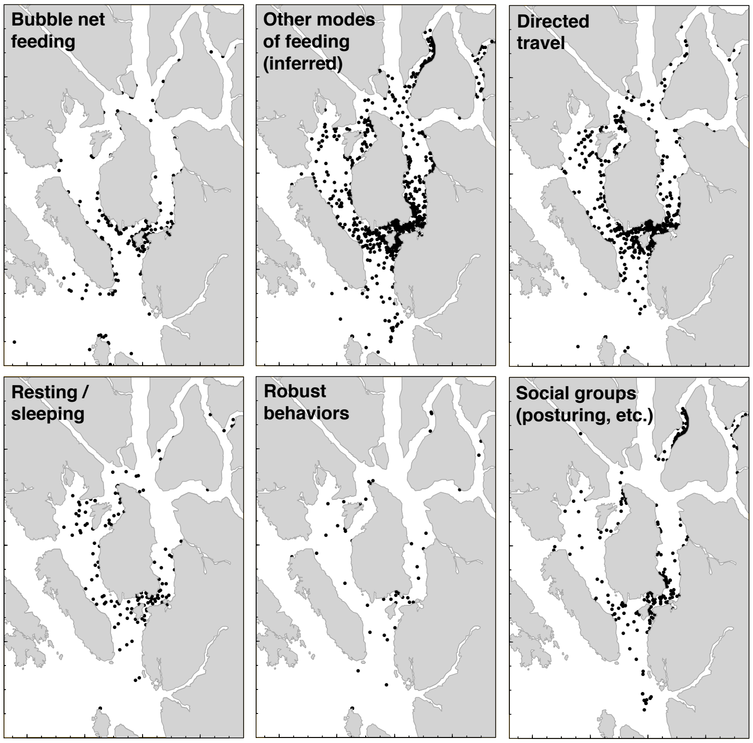

Figure S4. Locations of observations of various behaviors exhibited by humpback whales in the Kitimat Fjord System. See main text for detailed descriptions of behaviors.

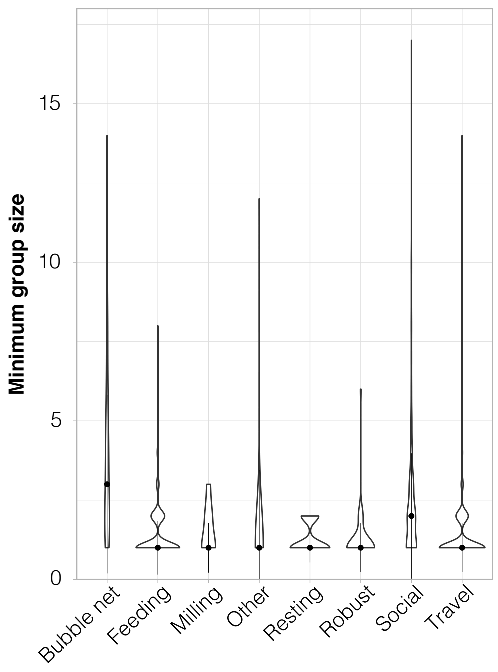

Figure S5. Violin plot of minimum humpback group sizes within each predefined behavior categories (n=8). Black points and associated bars indicate the median +/- one standard deviation. All categories except bubble-net feeding show higher sighting densities around 1 (tested using GLMM, Table S1.).

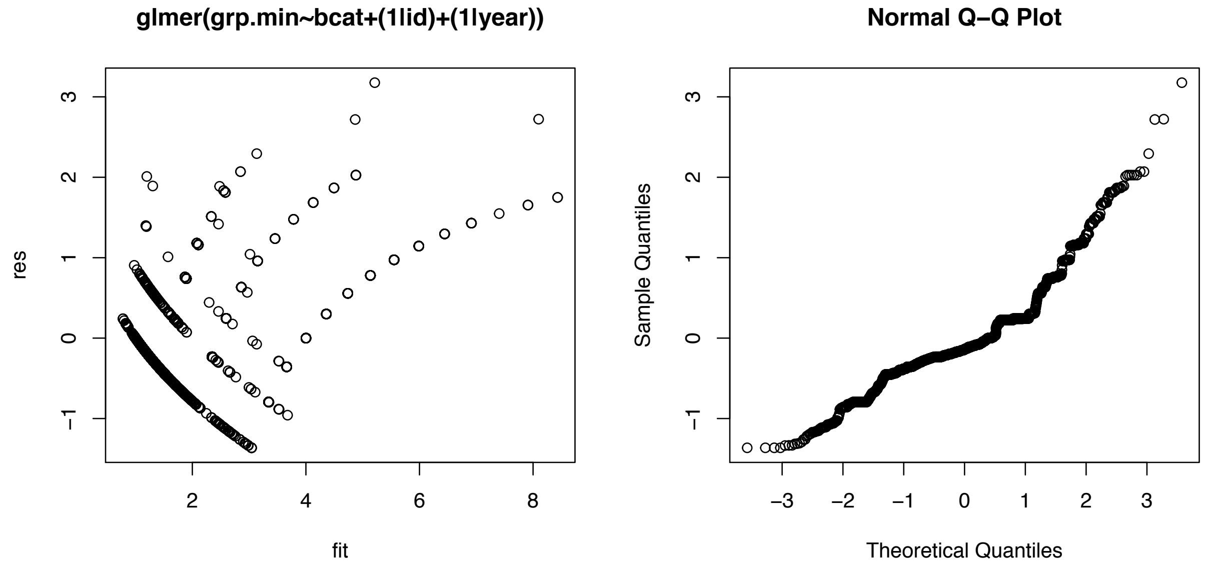

Figure S6. Diagnostic plots of Poisson GLMM model, testing fixed effects (behavior category) on minimum observed group size. Crossed random effects were individual identity (‘id’) and sighting year. As expected with a Poisson distributed GLMM, residuals are clustered around zero and one.

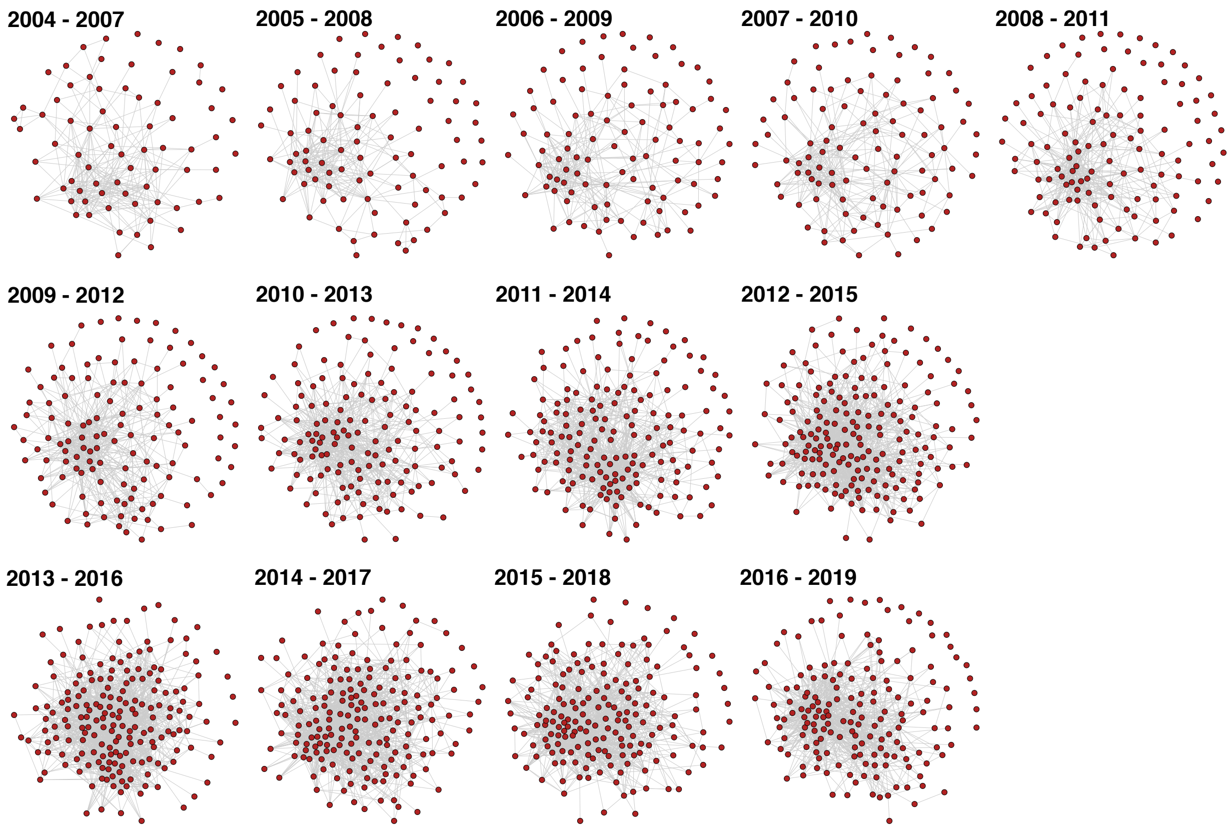

Figure S7. Annual time series of the association network, based on a four-year running window (2004-2007, 2005-2008, …, 2016-2019; n=12) for the population of humpback whales seen on at least five occasions throughout the 16-year study.

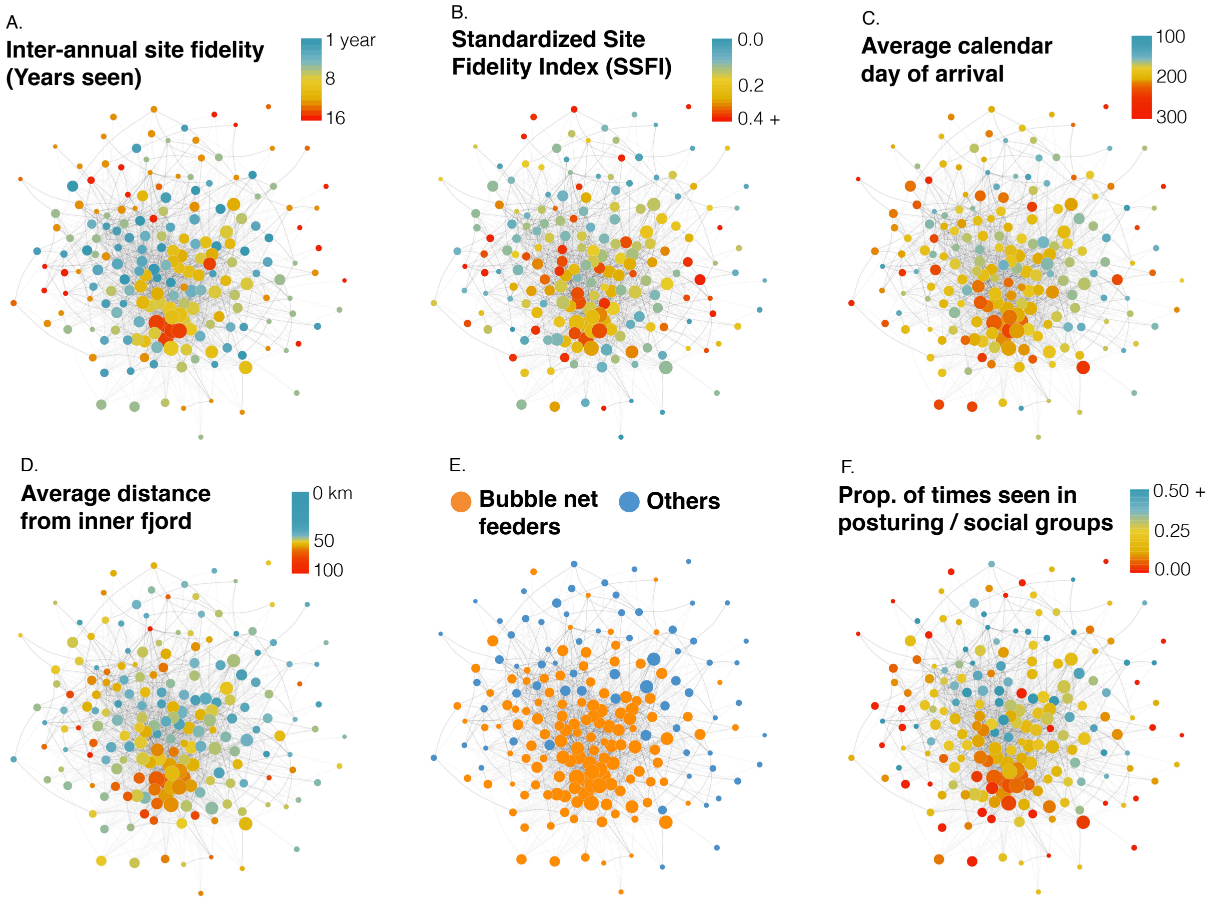

Figure S8. Association network of humpback whales (>= 5 encounters; n_{edges} = 19,306; n_{whales} = 196) based upon the Simple Ratio Index and color-coded by various aspects of habitat use and behavior. In all networks the placement of individuals remains the same, demonstrating related patterns in the distribution of individual traits. Vertex size reflects the number of observations of each individual. Thicker, darker edges represent statistically significant associations based upon data stream permutations (1,000 iterations). Networks built with igraph in R using the Fruchterman-Reingold layout.

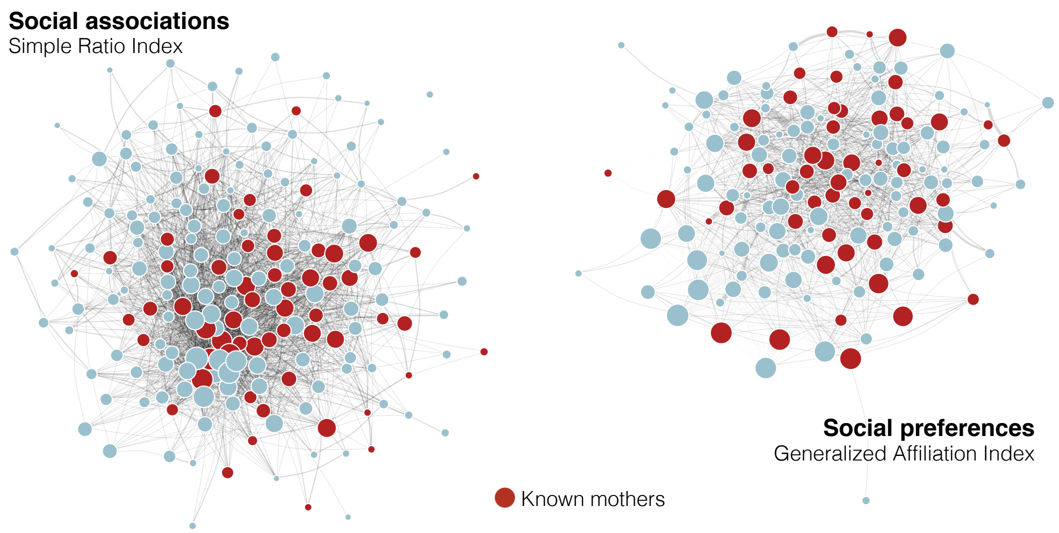

Figure S9. Social networks of humpback whales (>= 5 encounters) highlighting known mothers in red. The left network is based upon social associations (weighted by the Simple Ratio Index); right network includes the network of social preferences (weight by the Generalized Affiliation Index). Vertex size reflects the number of observations of each individual. Thicker, darker edges represent statistically significant associations/preferences based upon data stream permutations (1,000 iterations). Networks built with igraph in R using the Fruchterman-Reingold layout.

REFERENCES

1. White GC, Garrott RA. Analysis of Wildlife Radio-tracking Data. California: Academic Press: San Diego; 1990.

2. Whitehead H. Investigating structure and temporal scale in social organizations using identified individuals. Behav Ecol. 1995;6: 199–208.

3. Perryman RJY, Venables SK, Tapilatu RF, Marshall AD, Brown C, Franks DW. Social preferences and network structure in a population of reef manta rays. Behav Ecol Sociobiol. 2019;73: 114.

4. Whitehead H. Analyzing animal societies: quantitative methods for vertebrate social analysis. University of Chicago Press; 2008.

5. Farine DR. A guide to null models for animal social network analysis. Methods Ecol Evol. 2017;8: 1309–1320.

6. Whitehead H. SOCPROG: programs for analyzing social structure. 2019. Available: http://whitelab.biology.dal.ca/SOCPROG/Manual.pdf

7. Whitehead H. Analysis of animal movement using opportunistic individual identifications: application to sperm whales. Ecology. 2001;82: 1417–1432.

8. Burnham KP, Anderson DR. Model selection and multimodel inference, 2nd ed. A practical information-theoretic approach. New York: Springer; 2002.

9. Whitehead H. Selection of Models of Lagged Identification Rates and Lagged Association Rates Using AIC and QAIC. Communications in Statistics - Simulation and Computation. 2007;36: 1233–1246.

10. Tschopp A, Ferrari MA, Crespo EA, Coscarella MA. Development of a site fidelity index based on population capture-recapture data. PeerJ. 2018;6: e4782.

11. Acevedo J, Mora C, Aguayo-Lobo A. Sex-related site fidelity of humpback whales (Megaptera novaeangliae) to the Fueguian Archipelago feeding area, Chile. Mar Mamm Sci. 2014;30: 433–444.

12. Bolker BM, Brooks ME, Clark CJ, Geange SW, Poulsen JR, Stevens MHH, et al. Generalized linear mixed models: a practical guide for ecology and evolution. Trends Ecol Evol. 2009;24: 127–135.

13. Zuur AF, Ieno EN. A protocol for conducting and presenting results of regression-type analyses. Freckleton R, editor. Methods Ecol Evol. 2016;7: 636–645.

14. Bates D, Maechler M, Bolker B, Walker S. Fitting Linear Mixed-Effects Models Using lme4. J Stat Softw. 2015;67: 1–48.

15. Wray J, Keen EM. Calving rate decline in humpback whales (Megaptera novaeangliae) of northern British Columbia, Canada. Mar Mamm Sci. 2020;36: 709–720.

16. Farine DR. Animal social network inference and permutations for ecologists in R using asnipe. O'Hara RB, editor. Methods Ecol Evol. 2013;4: 1187–1194.

17. Whitehead H, James R. Generalized affiliation indices extract affiliations from social network data. Methods Ecol Evol. 2015;6: 836–844.

18. Godde S, Humbert L, Côté SD, Réale D, Whitehead H. Correcting for the impact of gregariousness in social network analyses. Anim Behav. 2013;85: 553–558.

19. Dekker D, Krackhardt D, Snijders TAB. Sensitivity of MRQAP Tests to Collinearity and Autocorrelation Conditions. Psychometrika. 2007;72: 563–581.

20. Hastie TJ, Pregibon D. Chapter 6. Statistical models in S Generalized linear models Ed by JM Chambers and TJ Hastie, Wadsworth and Brooks/Cole. 1992.

21. Whitehead H. SOCPROG programs: analysing animal social structures. Behav Ecol Sociobiol. 2009.

22. Csardi G, Nepusz T. The igraph software package for complex network research. InterJournal, Complex Systems 1695. 2006. Available: http://igraph.org

23. Blondel VD, Guillaume J-L, Lambiotte R, Lefebvre E. Fast unfolding of communities in large networks. J Stat Mech: Theory Exp. 2008;2008: P10008.

24. Newman MEJ. Finding community structure in networks using the eigenvectors of matrices. Phys Rev E Stat Nonlin Soft Matter Phys. 2006;74: 036104.
